# ECG Standards and Formats for Interoperability between mHealth and Healthcare Information Systems: A Scoping Review

**DOI:** 10.3390/ijerph191911941

**Published:** 2022-09-21

**Authors:** Daniel Cuevas-González, Juan Pablo García-Vázquez, Miguel Bravo-Zanoguera, Roberto López-Avitia, Marco A. Reyna, Nestor Alexander Zermeño-Campos, María Luisa González-Ramírez

**Affiliations:** Facultad de Ingeniería, MyDCI, Universidad Autónoma de Baja California, Mexicali 21100, Mexico

**Keywords:** ECG, EKG, interoperability, standards, mobile health systems, healthcare information systems

## Abstract

Interoperability is defined as the ability of a system or device to communicate between different technologies and software applications. This allows the exchange and use of data in an efficient, precise, and robust way. The present article gives researchers and healthcare information systems developers a qualitative and quantitative synthesis of the state of knowledge related to data formats and data standards proposed for mHealth devices interoperability in healthcare information systems that retrieve and store ECG data. We carry out a scoping review to answer to following questions: (1) What digital data formats or data standards have been proposed for the interoperability of electrocardiograph data between traditional healthcare information systems and mobile healthcare information systems? (2) What are the advantages and disadvantages of these data formats or data standards? The scoping review was conducted in four databases in accordance with the JBI methodology for scoping reviews, and in line with the Preferred Reporting Items for Systematic Reviews and Meta-Analyses extension for Scoping Reviews (PRISMA-ScR). A total of 4018 studies were identified of which 30 studies met the inclusion criteria. Based on our findings, we identify four standards and nine formats for capturing and storing streaming ECG data in mobile health applications. The standards used were HL7, SCP-ECG, x73-PHD, and PDF/A. Formats include CSV, PDF-ECG, and seven XML-based formats. These are ECG-XML, HL7-XML, mPCG-XML, mECGML, JSON, SaECG, and CDA R2.

## 1. Introduction

Over the years, technological development has come to reshape the way humans carry out daily activities. For instance, nowadays people are able to conduct financial activities with a single tap on their smartphones and commute from one city to another without memorizing a map. In addition, people can wear wearable devices that are designed to monitor health or even provide interventions to modify their health status. One approach is called mobile health or mHealth [1]. mHealth solutions are on the rise, the mHealth market was valued in 2020 at USD 22 billion and should reach USD 213.6 billion by 2025 [2]. However, designers and developers of mHealth applications face many challenges, such as usability, data security and confidentiality, network access, system reliability, as well as its integration (interoperability) [3].

Interoperability, in the context of healthcare information systems (HIS) is defined as the ability of a system or device to communicate between different technologies and software applications. As a result, data exchange and use can be carried out efficiently, accurately, and robustly [4].

In that way, interoperability in healthcare information systems is essential, as it facilitates the exchange of data between systems and its analysis and interpretation. This leads to lower costs, better patient care, and enhanced health care outcomes [5,6]. Furthermore, interoperability is seen as a precondition for digital innovations that could accelerate medical progress, allowing the use of artificial intelligence and big data [6].

In order to understand interoperability in health care information systems, there are several frameworks that have been proposed in the literature [7,8,9]. In this article, we have adopted the one proposed in Whitman [10], this is due to how interoperability is classified when we take into consideration the approach to the use of information and communications technologies in healthcare.

Therefore, four levels are proposed: (1) *Technical*. This type of interoperability often focuses on communication protocols and the necessary infrastructure to operate. (2) *Syntactic*. This is usually related to data formats used to transfer messages between systems. (3) *Semantic*. This interoperability level is associated with the content’s meaning. It refers to the human interpretation of the content, rather than the machine’s interpretation. Therefore, interoperability at this level means that there is a common understanding among people about the meaning of the content (information) that is being exchanged. (4) *Organizational*. As its name suggests, it is the ability of organizations to communicate and effectively transfer data, even though a variety of different information systems based on very different infrastructures are being used through different means.

In the literature, a number of studies offer mHealth applications that use different data formats for storing information that do not offer interoperability [1,6,11,12].

### ECG Data Interoperability

Despite the fact that diagnostic procedures in medicine have advanced significantly, certain techniques for identifying and treating diseases are still employed today. The electrocardiogram (ECG) is the most commonly employed diagnostic tool for heart disease detection [13]. It has been in use for over a century and, as a result, there are numerous data formats and electrocardiography equipment, representing an interoperability problem in health information systems.

In the literature, there are reviews that provide researchers with a synthesis of the different data formats or data standards that facilitate the interoperability between systems that retrieve and store electrocardiographic (ECG) data. For instance, in Bond et al. [14] present a review of ECG data in 10 formats. The studied data formats are SCP-ECG (Standard Communications Protocol for Computer-Assisted Electrocardiography), DICOM-ECG (Digital Imaging and Communication in Medicine), HL7 (Health Level Seven), aECG (Annotated Electrocardiogram), ecgML (a markup language for electrocardiogram data acquisition and analysis), MFER (Medical Waveform Format Encoding Rules), Philips XML (eXtensible Markup Language), XML-ECG, mECGML (mobile Electrocardiography Markup Language), and ecgAware (an ECG markup language for ambulatory telemonitoring and decision-making support). The data formats of SCP-ECG, DICOM-ECG, HL7, and aECG were analyzed in detail due to being considered predominant in the literature. In addition, the authors did a SWOT analysis to identify these data formats’ strengths, weaknesses, opportunities, and threats regarding interoperability. It was concluded that binary and XML data formats are the most suitable for encoding an ECG signal for use by a healthcare information system. However, the most accepted by the medical industry has been XML (eXtensible Markup Language). Finally, they conclude that a lack of global interoperability of ECG information remains. In the same way, in Baldini et al. [15] introduced a review of the most commonly used data formats for digital ECG. These data formats are linked with DICOM, HL7, and SCP-ECG. Furthermore, another review presented by Trigo et al. [12] suggests different ECG data formats and open data standards. In addition, they explain the relationship between the data formats and present a reflection on the status of interoperability. In their results, they report 39 identified data formats. They were divided into seven groups. The groups are the following: Standard Development Organizations (SDOs), binary formats, XML formats, database formats, Integrating the Healthcare Enterprise (IHE) profiles, ontology-based formats, and proprietary formats. The authors argue the need to create a single standard for storage and reading to achieve interoperability. However, this can only be achieved through political commitment and international cooperation between the various standardization bodies. Finally, Olamidipupo et al. [16] present a review of standards developed to facilitate seamless data exchange related to ECG in wireless devices.

In the literature reviews previously described, we identified a wide variety of standards and formats that can be used for traditional monitoring and storing ECG signals. Some of these standards are XML, binary-based, or text formats that provide effective solutions for data compression or human readability [12,14]. However, none of these have been designed for mobile health (mHealth) [17], where the support of ECG data streaming is needed for acquisition and storage [18,19,20].

Further, considering that previous literature reviews were conducted about a decade ago, we consider that a new literature review is needed in this context. Therefore, in this article we present a scoping review, which is a literature review that helps to examine the emerging evidence of a body of literature on a given topic and give a clear indication of the volume of studies available as well as a broad or detailed overview of its focus [21]. The aim of our scoping review is to provide researchers with a qualitative and quantitative synthesis of the state of knowledge related to formats or standards that are proposed to tackle the challenge of interoperability of mobile healthcare information systems.

## 2. Materials and Methods

This scoping review was conducted in accordance with the JBI methodology for scoping reviews [22], and in line with the Preferred Reporting Items for Systematic Reviews and Meta-Analyses extension for Scoping Reviews (PRISMA-ScR) [23]. The scoping review was registered in the Open Science Framework registry (https://osf.io/us2bv; registered on 30 April 2022).

### 2.1. Research Questions

In order to develop the research questions, we define the following concepts related to population or participants/concept/context (PCC) that is recommended by the framework of JBI (see Table 1).

**Table 1:** Definition of Population, Concept, and Context.

Category	Include	Exclude
Population	Any human population	Non-human populations
Concept	Data format or data standard to manage or storage ECG patient information	Data formats or data standards for manage or storage other kind of human biosignals (e.g., EEG, GRS)
Context	Mobile health or healthcare systems	Not applicable

The research questions that guide this scoping review are:

1. What digital data formats or data standards have been proposed for the interoperability of electrocardiographic data between traditional healthcare information systems and mobile healthcare information systems?
2. What are the advantages and disadvantages of these data formats or data standards?

### 2.2. Data Sources and Search Strategy

To determine the appropriate studies, a search of the following academic research databases: Pubmed, ACM (Association for Computing Machinery), IEEE Xplore, and Scopus was conducted. Thus, these databases were selected on the basis that they contain journals with a medical engineering or computational approach (e.g., Journal of Biomedical Engineering, Transactions of Computing for Healthcare).

To conduct the search, a collection of the Medical Subject Headings (MeSH) database of the National Center for Biotechnology Information (NCBI) web portal related to the words: *mhealth* and *electrocardiography* were used. These terms were grouped into two blocks: (1) *technological approach*: personal health device, mobile Health, mHealth, telehealth, eHealth, mobile-health, digital health, telemedicine, wearable devices, and health information interoperability; (2) *area of interest*: ECG, EKG electrocardiography, digital electrocardiography, standard, standardization, format, and data schema. Subsequently, the terms were grouped to create a generic string, using Boolean operators such as AND and OR. Next, the generic strings were adapted considering the search guides of the different databases. The modifications consisted of using filters in each database, such as language, search dates, and the type of document. Lastly, a total of nine generic searching strings were generated. An example of a generic search is (ECG OR electrocardiography OR EKG OR “digital electrocardiography”) AND (standard OR standardization OR format OR data schema) AND “wearable devices” OR (“Health information” AND interoperability). All generic search strings adapted for each digital library are listed in Appendix A.

### 2.3. Eligibility and Exclusion Criteria

To select the studies, we defined the following eligibility and exclusion criteria.

#### 2.3.1. Eligibility Criteria

- We selected studies published in the English language in indexed journals or conference articles between 1 January 2009 and 30 April 2022.
- Studies that present or describe devices and systems that store and retrieve electrocardiographic (ECG) signal data in a data format that allows interoperability between personal medical devices and/or health information systems (HIS).
- Studies that demonstrate the interoperability of data standards or data formats for storing and retrieving ECG data.

#### 2.3.2. Exclusion Criteria

- We have excluded studies that present devices and systems that store and retrieve other biological or physiological data different from the ECG in a format that permits interoperability.
- Studies that report the use of formats or standards for storing electrocardiographic signals but do not demonstrate the interoperability of medical data in health information systems (HIS).
- Studies published and/or reported in documents not disseminated through ordinary commercial publication channels pose access problems (e.g., theses, research reports, book chapters, patents). Studies of this type are commonly called gray literature.

### 2.4. Study Selection

**Figure 1:**
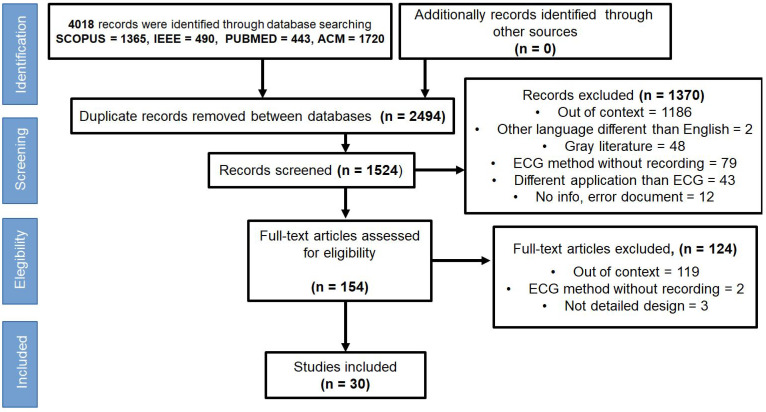
PRISMA-ScR flow diagram of the literature screening and selection process.

Figure 1 shows the study selection process reported using a Preferred Reporting for Systematic Reviews and Meta-Analyses extension for Scoping Reviews (PRISMA-ScR). In this section, the flowchart of the stages that were carried out for the selection of studies. The first stage was the identification. This was done by three authors (D.C.-G., J.P.G.-V., and N.A.Z.-C.) who consolidated the results of the searches run in the different databases. The second stage, called review, was conducted by two authors (D.C.-G. and J.P.G.V) who eliminated studies retrieved from more than one database to exclude duplicates. The third stage, selected, was carried out by the first and second authors (D.C.-G. and J.P.G.-V.) who screened the titles and abstracts of the articles to exclude those that did not meet the eligibility criteria. Disagreements about the study eligibility of the sampled articles were discussed between the two reviewers until a consensus was reached. To achieve it, they used a tool for systematic review support software called Rayyan (https://rayyan.qcri.org, accessed on 19 July 2022). This is a web or mobile device application that assists the researcher in peer reviewing titles and abstracts, allowing to exclude those that do not meet the eligibility criteria and identify and resolve discrepancies [24]. Finally, the stage called included consists of analyzing, in detail, all those studies that did meet the eligibility criteria.

### 2.5. Data Extraction

After performing the full-text reading of the selected articles, the relevant information was extracted by two independent researchers (D.C.-G. and J.P.G.-V.) using a personalized Google Form. Once the information has been extracted, we analyzed it in order to answer the research questions. We categorized the information as follows: data formats and standards used for interoperability between mHealth and healthcare information systems, as well as their advantages and disadvantages.

Data was gathered using a Google Form and organized into three categories: (1) data related to the reference: title of the article, year of publication, type of article, name of the journal or conference; (2) information related to the standard or format used in the study: data format used for the storage of ECG signals, data standards, protocols, scheme associated with the proposed one, characteristics of the format reported in the study, advantages of the format reported in the study, limitations of the format reported in the study; (3) information related to the analyzed study: objective of the study, device and/or sensor used to collect the ECG signal and results.

## 3. Results

### 3.1. Searches

A total of 4018 studies were identified through the searches; 2494 of which were eliminated due to being duplicated between the databases. The titles and abstracts for these 1524 studies were screened and, 1370 studies were irrelevant and therefore excluded. The remaining 154 studies were selected for further assessment of the full-text assessment. Among these, 124 studies were excluded due to the following reasons: studies that are not in context (*n* = 120); studies that analyze ECG signals without providing storage (*n* = 2); studies that only design a system (*n* = 3). Lastly, the final search yielded 30 studies that were included in this scoping review. A PRISMA-ScR flow diagram showing the study selection at each stage is detailed in Figure 1.

### 3.2. Study Characteristics

The studies selected for analysis (*n* = 30) were categorized by their publication year, type and subject area.

**Figure 2:**
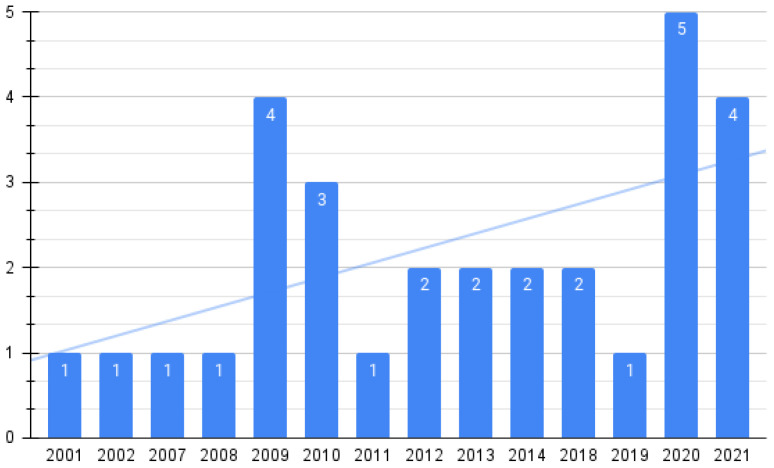
Studies published per year.

By publication year, in Figure 2 it is shown that the trend of studies reporting formats, standards, or even systems for format conversion to achieve interoperability in health information systems has been increasing since 2001.

**Figure 3:**
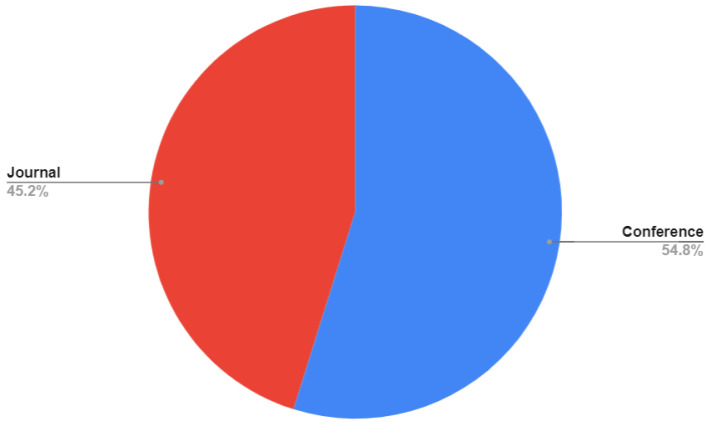
Type of article.

Regarding the type, in Figure 3 it is shown that 12 studies (40%) were published in journals and 18 (60%) on conference proceedings.

By subject area, 7 (23.33%) studies were from bio-engineering, 5 (16.67%) from computer science/medicine, 5 (16.67%) from computer science, 4 from computer science/medicine (13.33%), 2 from electrical engineering/medicine (6.66%), 2 from medicine (6.66%), and finally 1 from bio-engineering/computer science (3.33%). We also found an increasing rate in the number of published articles since 2001, with some gaps between 2004 and 2006 and 2015 and 2017.

### 3.3. Data Standards

Information standards are designed to ensure that all parties use the same language and approach when sharing, storing, and interpreting information [25]. In healthcare, data standards make up the backbone of interoperability [25].

Table 2 shows the standards identified. Of a total of eight standards identified in the documents, only four allow interoperability between healthcare information systems and mHealth systems.

#### 3.3.1. HL7

The Health Level 7 (HL7) is a medical standard that provides information to the medical healthcare ecosystem, allowing electronic patient records to be easily accessed, available, and understandable by the users [26]. Therefore, this has the ability to be used as an interchange standard alone or used in combination with other existing standards [26]. Consequently, HL7 outlines a well-defined framework for exchanging and integrating health information between different medical systems and healthcare organizations [26]. An HL7 message may contain ECGs, waveforms, measurements, a computer waveform analysis, and demographic information [27,28].

The healthcare industry has introduced many standard versions of HL7. Being firstly introduced in 1987, v1.0 was followed by different versions of v2, then v3, and finally FHIR, a new specification based on emerging industry approaches and experiences from v2 and v3 [29,30].

The advantages of the standard are that it not only provides data management and integration, but also provides machine-based processing while supporting the clinical decision system, as well as data standardization and structuring [30].

In contrast, the disadvantage of the HL7 standard is that it is not compatible with all medical devices. For example, the v2.x version is not compatible with v3.x and the v3 version is for clinical interface specialists; retooling and retraining is necessary; it is difficult to read HL7 messages unless developers are skilled enough due to delimiters; similarly, HL7 is not a complete package for medical image exchange: the syntax is difficult; it is only designed for the hospital environment. Not to mention how expensive it is, as well as its difficulty to program [27].

Some examples of systems that use this standard are presented in [31] where they performed remote monitoring by combining body area sensor networks with the HL7 standard and the IP Multimedia Subsystem platform to be able to operate with other technologies and to create a quality relationship between the patient and the doctor. Similar to the design in [19], which controls the data exchange and interoperability of medical information. In [30] they propose a new perspective on accountability in HL7, taking into consideration the organizational constraints in order to be able to increase access rights, and a new HL7 XML format that supports a business information framework.

#### 3.3.2. SCP-ECG

The Standard Communications Protocol for Computer-assisted Electrocardiography (SCP-ECG) is a standard intended primarily for short-term diagnostic ECGs endorsed by the European Committee for Standardization (CEN) [32]. It is encoded in a binary format that details the information content and structure to be exchanged between digital ECG devices and ECG hosts [32]. Furthermore, as SPC-ECG has been used since the 1990s, a large number of studies have reported the use and adaptation of this standard in the state of the art [32,33,34,35]. Moreover, it was promoted by the European project “Open ECG” to be used as a standard to solve the problem of interoperability [30]. Due to all the reasons given above, a wide variety of experience in the use of the SCP-ECG standard can be found in literature over the last two decades [12,17,28,30,36,37,38]. In 1993, it turned into a standard supported by CEN and later by AAMI. In 2009, it was approved as an ISO 11073-91064:2009 standard [39]. The latest version published by CEN in 2020 [32], which now encompasses rest and stress ECGs, Holter recordings, and protocol-based trials. Recently, the latest version of the SCP-ECG standard has become an international standard as ISO/IEEE 11073-91064:2009, part of the x73 family [40]. Although the SCP-ECG is a popular protocol, supported by around 70% of the major manufacturers of ECG devices and recently approved as an international standard (ISO/IEEE 11073-91064:200), it gives no consideration to data security or privacy. SCP-ECG files must be adequately protected to avoid data leaking and malicious manipulation, which could result in patients’ embarrassment, wrong diagnosis, and legal actions against hospitals [41]. Among the advantages of this format, its file compression can reduce files up to 40 times smaller than DICOM, HL7, and XML formats. Its encoding is binary, therefore it can be easy to adapt to file conversion systems that seek interoperability. Similarly, it has wide support due to its long history and application in electrocardiography and the literature. Nonetheless, the disadvantages are that it is intended for short-term electrocardiography records; it is not human readable; the compression processes make it prone to errors, and it does not support continuous storage or streaming. In the literature, the use of the presented standard proves the interest of the scientific community and sets the SCP-ECG standard as one of the most widespread initiatives in the standardization of medical informatics [17,42]. Particularly, some of the features of this standard include ECG signal, patient data, and ECG administrative data, as well as measurement and interpretation results [42]. In [43], an x73 compliant real-time transmission of ECG signals were reported using an Agent ECG device where they send the records of the ECG to the manager. An SCP-ECG file can be generated and sent through eXtensible Markup Language (XML) to an EHR server for later consultations. An improvement to cover the transmission of large ECGs has been presented and discussed in [38]. The result of this process has proved that the x73-PHD Message Part-Store concept is a well-defined idea to manage metric stored data, including ECGs. In [44], the framework guarantees the specific requirements established by x73-PHD and SCP-ECG, which facilitates the standardized exchange of digital ECG formats within, or across, healthcare information systems. Lastly, the alignment of two related standards, SCP-ECG and x73-PHD (particularly 11073-10406-do02), and the mapping between them, has been investigated and discussed in [12].

#### 3.3.3. X73-PHD

The ISO/IEEE 11073 standard, also known as x73, targets health devices designed for personal use. The objective is to facilitate the health data exchange while providing plug-and-play real-time interoperability [45].

This standard is an optimization of the optimized exchange protocol (ISO/IEEE 11073-20601) that defines a common framework in the design of an abstract model of personal health data (includes, besides ECG, applications such as pulse oximeters, thermometers, scales, baumanometers) [14]. One of the versions of this family of standards is ISO/IEEE 11073-10406-ECG basic (1–3 leads) [46].

In a literature review presented in [45], it is presented that the standard enables the design and implementation of interoperable mHealth systems.

The x73-PHD standard is based on the Optimized Exchange Protocol (ISO/IEEE 11073-20601), which sets out a common framework for the development of an abstract model of accessible personal health data. This is related to the transport-independent transfer syntax, which is required to build logical connections across systems. Additionally, it also functions to provide the presentation capabilities and services needed to perform communication tasks. Furthermore, the x73-PHD standard defines device specializations for different medical devices (such as pulse oximeters, blood pressure, thermometer, or weighing scales). As well as it outlined as a model-based reference a well-defined object-oriented paradigm that ensures extensibility and reusability by establishing three different models: (1) Domain information model (DIM). The DIM characterizes an agent’s information as a configuration of objects with one or more attributes, describing the measurement data that are communicated to the manager, as well as the elements that control the behavior and report the status of the agent. (2) Service model. The service model provides access to the data primitives that are sent between the agent and the manager to exchange data defined in the DIM. These primitives include commands such as Get, Set, Action, and Event Reporting. (3) Communication model. The communication model supports the topology of one or more agents communicating through point-to-point connections with a single manager. The dynamic behavior of the system for each point-to-point connection is defined by a connection finite state machine (FSM), which defines the states and sub-states through which a pair of agents and managers pass, including states related to connection, association, and operation [47].

At the present, x73-PHD’s disadvantages can be found in the small number of x73-PHD and that, at the moment, only a small number of PHD configurations have been defined by x73-PHD. Although x73-PoC (point of care) determines the ECG profile, x73-PHD does not have an ECG profile at the moment, therefore an adaptation is necessary [12,43]. This adaptation creates the inherent domain information model (DIM), which configures the information structure required to establish a finite state machine (FSM) and allows any PHD to act as a communication agent to the x73-PHD [43]. To solve this problem, in [38,47], the authors use the SCP-ECG standard, which is closely related to the x73-PHD, where even the SCP-ECG standard has become an international standard as ISO/IEEE 11073 91064: 2009, part of the x73 family.

As of now, x73 provides an important advantage: it has an architecture that not only enables the interoperability of medical devices, but also guarantees their portability to other environments, situations, and use cases (geriatric and rehabilitation services, athlete monitoring, etc.); facilitates remote management (medical information, alarms, etc.), technologies (Bluetooth, ZigBee, RFID), and devices (PDAs, SmartPhones, microcontrollers, etc.). The continuous evolution of x73 allows the development of standard e-Health point-of-care (PoC) solutions while migrating to new ubiquitous use cases (u-Health) for microcontroller implementation, device multiplexing, remote management, and integration with real-time signals in application environments whose frameworks are yet to be defined.

### 3.4. PDF/A

The Electronic Document File Format For Long Term Preservation (PDF/A) standard is part of a family of standards that have become popular among portable devices (smartphones and computers) including medical equipment such as portable electrocardiographs and wearables [17].

The purpose of the PDF/A format is to represent electronic documents in a manner that preserves their static visual appearance over time. This is independent of the tools and systems used for creating, storing, or rendering the files. A PDF/A document attempts to maximize device independence, self-containment, and self-documentation. The standard has four versions [48]:

- PDF/A-1 (ISO 19005-1:2005) was published in 2005 and is based on PDF version 1.4.
- PDF/A-2 (ISO 19005-2:2011) was published in 2011 and is based on PDF version 1.7. This version extended the capabilities of PDF/A-1. The main new capability was to allow embedding of PDF/A compliant attachments.
- PDF/A-3 (ISO 19005-3:2012) was released in 2012. This version extended the capabilities of PDF/A-2. There is a new feature that allows files of any format to be embedded.
- PDF/A-4 (ISO 19005-4:2020) is based on PDF version 2.0. PDF/A-4 introduces the new PDF/A-4e conformance level that supports interactive 3D models for engineering workflows.

The main advantages of this standard are that it seeks the long-term preservation of files, i.e., it seeks the homogenization of digital data; it is small in size and can be read by humans. This standard allows access to the file independently of the software used without modifying the characteristics of the document.

The disadvantages for PDF/A-1, PDF/A-2, and PDF/A-3 include that (1) audio and video content and 3D artwork are forbidden; (2) JavaScript and executable file launches are prohibited; (3) all fonts must be embedded in order to be legally embeddable for unlimited, universal rendering; (4) color spaces must be specified in a device-independent manner; (5) encryption is disallowed; (6) the use of standards-based metadata is mandated. Some examples of systems that use this standard are presented in [17,49] a format derived from the PDF format called PDF-ECG. This is proposed by combining the benefits of a digital ECG and a standard graphical report. The PDF-ECG format provides, within a unique structure, a way to satisfy both the needs for a simple graphical report, accessible without the installation of specific proprietary software, and for delivering all the acquired information for further processing with specialized software. This format represents a hybrid with advantages such as ECG graphs that can be interpreted by humans and healthcare specialists and meeting the requirements of the HL7 aECG standard by having embedded aECG data compression in which aECG data can be extracted by standard software for data processing in algorithms. A tool is developed for the transformation of the GE MAC 600 ECG device records. It benefits the extraction of the patient’s clinical data while generating ECG graphs in PDF format. This can be accomplished directly from an Android smartphone device without requiring a proprietary software [50].

**Table 2:** Standards; Level interoperability L1: Technical, L2: Syntactic, L3: Semantic, L4: Organizational.

Studies	Data Standard	mHealth Interoperability	Level Interoperability
[17,19,30,36,38,49,51,52,53]	HL7	Yes	L1, L2, L3
[12,17,19,30,53]	DICOM	No	L1, L3, L4
[12,17,19,43,47,49,53]	SCP-ECG	Yes	L1, L2, L4
[17]	ISHINE	No	L1
[54]	PDF/A	Yes	L1, L3, L4
[12,43,47]	X73-PHD	Yes	L1, L2, L4
[55]	Open ECG Philips	No	L2

### 3.5. Data Formats

Data formats are the internal structure and encoding of a digital object, which allows it to be processed or presented in an accessible way. Usually, they are classified as open and closed. The open format is where the specifications of the software are available to anyone, free of charge. In this way, anyone can use those specifications in their own software, without any limitation on their reuse that may be imposed by intellectual property rights [56]. Regarding the close or proprietary format, it is the one in which its specifications are not publicly available and its reuse is limited [57].

In Table 3, we present the data formats identified in this scoping review. Nine formats were identified in the documents, seven of which are derived from XML.

**Table 3:** Formats.

Studies	Data Formats	mHealth Interoperability	Type Format
[17,38,42,50,58,59,60]	XML-ECG	Yes	Open
[61]	HL7-XML	Yes	Open
[27]	mPCG-XML	Yes	Open
[62,63]	Philips-XML	No	Propietary
[64,65]	ecgML	No	Open
[14,66]	mECGML	Yes	Open
[53,67]	JSON	Yes	Open
[17]	SaECG	Yes	Open
[49,68]	HL7 aECG	Yes	Open
[52,69]	CDA R2	Yes	Open
[67]	PDF-ECG	Yes	Open
[70,71,72]	MFER	No	Propietary
[73]	EDF	No	Open
[74]	CSV	Yes	Open
[75]	ECGWARE	No	Open

#### 3.5.1. XML

The eXtensible Markup Language (XML) has become a de facto data format standard for the storage, hierarchical presentation, and display of data in tree form, as well as the transmission of XML in TCP-IP protocol and Internet applications [76].

Moreover, XML offers simple, scalable, highly interoperable, and highly human-readable solutions, as well as seamless integration with machines, protocols, and application architectures [12,27,77].

The XML standard consists of a domain-independent interface for exchanging various types of medical information and a common platform for clinical transactions [27].

The limitations that XML presents are the following: firstly, it partially addresses the content issues of ECG data; secondly, it is uneasy to extend for ECG and unsupportive to PCG annotations; lastly, it does not speak the PCG “language” and creates barriers for clinicians and experts. In addition, it also presents implications at the moment of integration into healthcare architecture and applications [27]. At the same time, another limitation regarding the development of electronic 12-lead ECG diagnostics is the heterogeneity of 12-lead ECG data formats, such as Standard Communications Protocol (SCP) binary ECG and Extensible Markup Language (XML) text-based ECG, which are not capable of integrating medical data based on waveforms and images. In addition, ECG waveforms stored in SCP-ECG and XML-ECG files are compressed or encrypted by many vendor-specific 12-lead ECG instruments in clinical practice [42].

There are several works that propose to use the XML format to perform format converters, for example, in [77] the authors present an XML project, an XML-based middleware is designed to convert a variety of ECG formats into HL7 message formats and integrate them with an existing Picture Archiving and Communication System (PACS). It can be considered more as a storage facility than as a true processing environment. Another example is presented in [78], where the authors proposed a web-based ECG management system to facilitate sharing of XML-ECGs between general practitioners/technicians in rural centers and expert cardiologists. The Java-based system processes the XML-ECG uploaded by a GP/technician and renders ECG waveforms as Scalable Vector Graphics (SVGs) in a web browser to the cardiologist. However, the use of SVG has been criticized due to inherent limitations in XML encoding and transmission of large datasets along with slow response time to point-and-click activity [59].

On the other hand, in the literature we identified several XML-based formats that have been proposed to facilitate interoperability between mHealth systems and traditional healthcare information systems. Each of these formats is described below.

- mPCG-XML is a markup language specifically designed for the presentation, visualization, and transmission of PCG data and its seamless integration with telecardiology applications. Telemonitoring for remote access to PCG data and transmission of PCG data over the mobile network using mPCG-XML ensure data interoperability and support data mining and semantics.
  In order to ensure interoperability and support data mining and data semantics, the authors of [27] propose a new method that uses an XML schema exclusively for PCG data exchange and monitoring over mobile devices. This XML schema is called mPCG-XML, which provides fast medical decision assistance. Additionally, it supports a hierarchical structure that captures data, tags, and elements in an efficient way, so that it is human readable and will enable seamless integration of PCG data in healthcare architecture and applications.
- SaECG (Stream-enabled annotated ECG) is an XML-based format that allows the storage of long-duration ECG traces based on the FHIR (Fast Healthcare Interoperability Resources) standard specifications for the HL7 aECG format, however, adding annotations for the time period in which the measurements are taken and divided into several segments, taking into account the periods of sensor reading acquisition and inactivity periods [19].
  The advantages of the format are that SaECG is compatible with different ECG streaming sensors and is capable to use many and independent channels [19].
- JSON (JavaScript Object Notation) is a format defined at the end of 2002 by Douglas Crockford that emerged from the need for data exchange with web services, based on the data types of JavaScript language [79,80].
  Files generated from the JSON format are dictionaries that consist of a tree structure nested values identified by key-value pairs, thus supporting two types of data structures: arrays and objects. Branches may or may not have the same key values, allowing data to be standardized by tagging it with specific topics. In that way, data are identified and generated from different sources or further formats, while merging them into one [80,81].
  To navigate through a JSON document, the notation to be used will vary between systems without ignoring the following principles, previously described in [80]:
  - As a JSON object, a specific value is accessed by a key-value pair.
  - As a JSON array, a specific element must be accessed by the i-th element of the array.
  Among its main advantages are its data exchange with web services through the use of an API (Application Programming Interface) and the ease with which it can be interpreted by both humans and other systems, such as NoSQL or graph databases, due to its nested structure and key-value identifiers [80]. Since it reduces the data volume needed to identify each file element, it is also known to be lighter than other formats. This is due to its size reduction before being transmitted, allowing this format to be used by different programming languages and platforms. Therefore, it is more efficient in transmitting data between the different modules of the same application [79].
  However, one of its disadvantages when operating with other applications is the impossibility of specifying the data format, making it difficult to transmit files such as images [79].
- Philips XML was published in 2003, and is used by its own electrocardiographs, bedside monitors, and defibrillators. This facilitated the European Commission’s effort to ensure electrocardiograph interoperability and ECG accessibility. W3C XML Schema Language was used to write the Philips XML format, which was available on the Internet and included the electrocardiograph documentation. As part of the Philips XML ECG, the ECG waveform data is compressed using a lossless algorithm and encoded using a base 64 encoding scheme into ASCII characters. To facilitate the easy access to compressed waveform data, Philips also provides a suite of software tools. Furthermore, Philips’ XML format uses Scalable Vector Graphics (SVG) as a display format and is compatible with other standards and initiatives, such as HL7 aECG and Integrating the Healthcare Enterprise (IHE) for displaying ECGs.
- HL7 aECG is an XML-based standard for medical data digitization created by HL7 RCRIM (Regulated Clinical Research Information Management) which was accept in 2004 by American National Standards Institute, where HL7 aECG is a sub-standard that supports the storage and display of ECG data [28,43,51,54,77]. The format includes one or more time-bound ECG waveform data sets and annotations for that time. The message model is derived from HL7 RIM (Reference Information Model). The aECG R-MIM (Refined Information Model) forms the basis for defining messages and XML schema. Different ECG annotations can be defined with it (e.g., QRS wave, T-offset, P wave, peak R time, R peak amplitude, QT interval, QTc interval annotation, etc.). It supports a 12-channel ECG with a maximum sampling time of 30 seconds. Unfortunately, it does not support the ECG data stream [51].
- ecgML is another XML-based standard for presentation and storage of ECG and effective XML transformations using Extensible Stylesheet Language Transformation technology in various formats such as comma-separated files and scalable vector graphics (SVG) [27].
- XML-ECG The XML-ECG format was published in 2007. This standard uses only six modules, making it much more readable. Although the structure is simple, it can describe the complete ECG information, including waveform, patient demographics, annotation, and measurement, to name a few. In addition, it is also expandable with the explicit rule of separating the basic part and the expandable part in the structure [60].
- CDA R2 In May 2005, the Clinical Document Architecture Release two, became an ANSI-approved HL7 standard. CDA documents consist of text, images, sounds, and other multimedia content. It can be transferred within a message and can exist independently, outside the transferring message [82]. It is important to note that CDA documents are encoded in eXtensible Markup Language (XML) and they derive their machine-processable meaning from the Reference Information Model [82]. The CDA R2 document includes a document header and the document body. As a result, the document contains several sections that contain human-readable narrative forms or coded structures for automatic processing [83]. In CDA R2, health data can be easily integrated into databases of healthcare facilities.

#### 3.5.2. PDF-ECG

This is a hybrid ECG archival format that allows storing in the same file both the standard graphical report of an ECG together with its source ECG data (waveforms) [54]. This format presents the following advantages [54,84]: (1) ECG data portability, long-term archiving, and documentation; (2) the ECG signal is on a file format used to represent documents in a manner independent of application software, hardware, and operating systems.

#### 3.5.3. CSV

Comma-separated value (CSV) is a file that stores information in tabular format by sorting the values by columns that are separated by a comma [85], allowing the manipulation of data allowing a simple and compact manipulation of data, thus reducing the file size and efficiently providing the data [86,87,88].

Due to the simplicity of its format, it is often used to store records from sensors or contents of various health informatics data tables whose fields can be separated by columns and is widely used in areas of signal processing, natural language processing, and big data analytics [88,89,90].

The CSV format has several advantages, among them is its human readability since the data is not coded or labeled, but simply placed one after the other. In that manner, its manipulation is improved by almost any text editor or data analysis application. Its simplicity makes it a popular choice for number-crunching jobs. In addition, its structure offers memory savings by not handling labeling [87,88].

In contrast, the main disadvantage is its inability to store complex or hierarchical data, therefore it is widely unused among developers [87,91].

## 4. Discussion

In this article, we present a scoping review that aims to quantify and summarize the existing evidence of ECG formats and standards to enable interoperability between mHealth and health information systems.

In Figure 2, it is shown that 60% of the articles identified have been published in conferences. This suggests that this is an ongoing discussion, as interoperability is still a challenge for designers of health information systems, mainly for mHealth applications [92,93,94]. This can be seen in Figure 3, which shows that there is an increasing trend in the number of studies reporting standards.

Consequently, a total of thirty studies were analyzed to identify the ECG standards and formats that enable interoperability between mHealth and health information systems. We identify five standards and ten formats, listed in Table 2 and Table 3. The most common standard is HL7 and the most common format is XML.

Regarding standards and formats for interoperability, we identify that there has been an increased interest in the use of open formats and standards [34,49,95,96] since those standards and formats allow users not to be locked into a specific provider, which increases market flexibility. As a result, the market becomes more competitive for technologies and solutions complying with the formats and standards.

Regarding the level of interoperability in the standards: HL7’s advantages are on technical and syntactic levels, it is capable of integrating with other standards and/or protocols for the exchange of information. In semantic levels it complies partially, it is intelligible to the user, although it needs to be interpreted by a health specialist due to its complexity. Finally, organizational interoperability remains a challenge for this standard even between versions of the same standard. SCP-ECG complies with interoperability at the technical, syntactic, and organizational levels because it uses a binary format. This facilitates the exchange of information for conversion or processing by other systems, standards, and formats. However, a limitation of semantic interoperability is that it cannot be read by the user. x73-PHD has good technical, syntactic, and organizational interoperability due to its latest update that allows interoperability with medical devices and allows portability to other environments, situations, and use cases. However, this standard at the moment only has a small number of PHD configurations, presenting a limitation in semantic interoperability. PDF/A has technical, semantic, and organizational interoperability because it is compatible with practically any system regardless of the software used, and it can also be read by humans. However, it does not have syntactic interoperability because it operates independently of the tools and systems used to create, store, or render the files.

Finally, we have identified mHealth applications that are characterized by the use of sensors and wearable devices that users carry with them for constant monitoring. As these applications must process streams of biosignal data that can be visualized or stored, they cannot use current formats or standards because they do not provide the necessary support for these processes. Therefore, the use of novel formats based on open standards has been proposed, such as ECG-XML, JSON, ECG-PDF, HL7 aECG, SaECG, CDA R2, and CSV [17,19,34,51].

However, there are other conditions that add value to the selection of a format and this depends on the type of development envisaged, since efficiency is not only based on interoperability, but also on the management of computational resources such as RAM, CPU usage, transfer rate and query speed, which play an important role in the selection of the format [97,98]. This is because formats such as XML are shown as inflated files by placing start and end tags on each of their elements and increasing the size of their final file and JSON, despite being a format that requires less storage space due to its hierarchical structure, still uses a greater number of characters to identify its elements compared to the CSV format [19,99,100,101].

We consider that, in the future, one format that will be used to store large amounts of ECG signal samples measured by mobile devices will be DNA-type structures, which use four nucleotides that make up the genetic code: A, T, G, and C. For example, G and C could be used to represent 0 while A and T represent 1 [102]. This way of storing information has many advantages, such as it is extremely dense, with a raw limit of 1 exabyte/mm^3^ (109 GB/mm^3^), and long-lasting, with an observed half-life of over 500 years [103].

Nonetheless, a limitation of this work is that we only selected journal or conference articles published in English.

## 5. Conclusions

Our findings provided engaging information that forthcoming mHealth device developers could consider useful. Health system information designers could also benefit from it to understand the interoperability problem due to the diversity of formats and standards. Nowadays, interoperability continues to find challenges in the health system information of ECG recordings, which is caused by a lack of device homogeneity, but is also due to the existence of numerous standards and formats.

From the findings of this review, we can identify that there is a preference for the format to be used, depending on the purpose for which the data will be put. Furthermore, the scientific community is inclined to formats such as CSV when processing the collected records to obtain statistical describing information. These formats are easily manipulated by data visualization and applications such as Excel, Tableau, PowerBI, or even text editors. In contrast, for the development of user interfaces to display recorded data, there is an inclination to formats such as XML and JSON, which allow the nesting of information. It also benefits from the addition of additional findings tags or data. Due to the latter, specific indications can be placed for the software or application that will read the information and present it to the end user.

Therefore, the investigation of this topic continues to be a relevant topic for the scientific community. New formats based on open standards were identified in the last decade that have not been reported in previous reviews. In addition, an evolution in the most used formats and standards for conventional and mHealth electrocardiography needs could be considered to propose a homogenized solution, in order to achieve interoperability in health information systems.

## Data Availability

Not applicable.

## Appendix A. Search Strategy

A list of the generic and adapted search strings can be found in the following document: https://goo.su/qPUpVy (accessed on 30 April 2022 ).
